# Restenosis is associated with prothrombotic plasma fibrin clot characteristics in endovascularly treated patients with critical limb ischemia

**DOI:** 10.1007/s11239-019-01826-9

**Published:** 2019-02-14

**Authors:** Tomasz Nowakowski, Krzysztof Piotr Malinowski, Rafał Niżankowski, Teresa Iwaniec, Anetta Undas

**Affiliations:** 1 Department of Angiology, Jagiellonian University Medical College, 8 Skawinska St, 31-066 Krakow, Poland; 2 Krakow Cardiovascular Research Institute, Krakow, Poland; 3 Institute of Public Health, Faculty of Health Science, Jagiellonian University Medical College, Krakow, Poland; 4 Department of Medicine, Jagiellonian University Medical College, Krakow, Poland; 5 Institute of Cardiology, Jagiellonian University Medical College, Krakow, Poland; 6 John Paul II Hospital, Krakow, Poland

**Keywords:** Fibrin clot, Peripheral arterial disease, Endovascular treatment, In-stent restenosis

## Abstract

**Introduction:**

Hypolysible fibrin clots composed of tightly packed fibers characterize patients with peripheral artery disease (PAD) especially those with critical limb ischemia (CLI). Little is known about the impact of a prothrombotic clot phenotype on restenosis following endovascular revascularization in CLI. The goal of this study was to compare fibrin clot properties and their determinants in CLI patients with restenosis after endovascular treatment (ET) and those free of this complication.

**Methods:**

85 patients with CLI and restenosis within 1 year after ET on optimal pharmacotherapy and 47 PAD control patients without restenosis were included into the study. Plasma fibrin clot permeability (Ks, a measure of the average pore size in the fibrin network) and clot lysis time (CLT) with its potential determinants were determined. During follow-up, the composite endpoint including re-intervention, amputation and death was assessed.

**Results:**

Compared with the control group, patients with restenosis had reduced K_s_ (− 9.5%, *p* < 0.001), prolonged CLT (+ 12.4%, *p* = 0.003), higher thrombin generation (+ 7.9%, *p* < 0.001) and elevated von Willebrand factor (vWF) antigen (+ 14.2%, *p* < 0.001). During a 24 months follow-up the composite endpoint occurred in 54 CLI patients with restenosis (63.5%) and nine control patients (19.1%, *p* < 0.001) with no association with baseline K_s_ and CLT.

**Conclusion:**

The increased thrombin formation and unfavorable fibrin clot properties occur in patients with CLI who experienced restenosis despite optimal endovascular and pharmacological therapy.

**Electronic supplementary material:**

The online version of this article (10.1007/s11239-019-01826-9) contains supplementary material, which is available to authorized users.

## Highlights

- Plasma fibrin clot permeability and clot lysis time are non-standard but clinically useful tools for atherothrombotic risk assessment.
- CLI patients with restenosis after endovascular treatment demonstrate unfavorable clot characteristics.
- The association of prothrombotic clot features and poor outcomes in endovascularly treated CLI patients is not evident.
- Prothrombotic clot phenotype might be modified by tailored pharmacotherapy especially anticoagulation which should be evaluated in future studies in this group of patients.

## Introduction

Peripheral artery disease (PAD) is a common age-dependent disease with its prevalence up to 18–20% in patients over 70 years of age. The prevalence of intermittent claudication is 3–7% of patients and critical limb ischemia (CLI) the most advanced form of PAD, associated with a drastic limitation of quality of life, amputation, increased cardiovascular events and mortality occurs in 1–3% of patients with PAD with a likely rising prevalence in the future [1–5]. The 1 year incidence of major amputations (above the ankle), as well as MI or stroke in patients with CLI without revascularization reaches 30–50% and death occurs in up to 25% in the five following years [1, 6].

The most effective treatment of CLI is surgical or endovascular revascularization of the endangered limb, with similar rates of amputations, amputation-free survival and mortality [7–9]. Known factors that improve outcomes of ET in CLI involve drug eluting stents, drug eluting balloons, endovascular atherectomy, cutting balloons and retrograde tibiopedal access [4, 10, 11]. It is unclear whether any hemostatic parameters may contribute to outcomes of such therapy.

Enhanced blood coagulation linked to the systematic inflammatory state, reflected among others by elevated plasma fibrinogen, is implicated in PAD and its complications [12]. The conversion of plasma fibrinogen into fibrin and fibrin clot formation constitute the final step of the blood coagulation. Fibrinogen and fibrin promote plaque growth [13]. Numerous environmental and genetic factors influence fibrin clot characteristics and plasma fibrinogen concentrations and its function are of key importance [14]. Of note, most PAD risk factors such as diabetes, arterial hypertension, cigarette smoking, have been demonstrated as unfavorable modulators of fibrin clot properties [15].

It has been shown that dense fibrin networks composed of thin and tightly packed fibers with relatively reduced susceptibility to lysis characterize PAD [16]. Altered fibrin clot structure and function were found in relatively young individuals with intermittent claudication, in the healthy first-degree relatives of patients with claudication and in premature PAD patients [17–19]. In 2011 we reported unfavorable fibrin clot properties also in patients with advanced PAD especially in those with worse clinical outcomes during 5 years of follow-up [20].

Until now there have been no reports assessing the impact of the prothrombotic clot phenotype on restenosis following endovascular revascularization in CLI. We hypothesized that PAD patients with CLI who experienced restenosis despite optimal endovascular and pharmacological treatment characterize more prothrombotic fibrin clot properties involving denser fiber meshwork relatively resistant to lysis compared with those with good outcomes at a 1 year follow-up.

## Patients and methods

Among 697 patients with CLI defined according TASC criteria [1], treated endovascularly at the Department of Angiology of the Jagiellonian University in Cracow between February 2014 and February 2016, we screened and enrolled 140 consecutive patients in whom symptomatic restenosis in the treated segment occurred over the 12 month follow-up period.

Exclusion criteria were: recent (< 6 months) deep vein thrombosis, pulmonary embolism, acute coronary syndrome or cerebrovascular episode, known malignancy, signs of acute infection, end-stage renal failure and oral anticoagulant therapy (International Normalized Ratio [INR] > 1.5). Patients with premature cessation of antiplatelet drugs (n = 17), with suboptimal result of the percutaneous angioplasty (PTA) during the primary procedure (n = 12) and those with incomplete laboratory data (n = 5) were also excluded.

The control group comprised 47 PAD outpatients with similar cardiovascular risk factors treated percutaneously (initially 32 CLI patients and 15 non CLI patients), who demonstrated improvement in limb ischemia, pain relief and no symptoms of restenosis in the last 12 months after PTA.

CAD was defined as a history of MI, coronary intervention, or hospitalization for angina symptoms. Diabetes was diagnosed based on the use of insulin or oral hypoglycemic agents. Cerebrovascular disease was defined as a documented history of stroke or transient ischemic attack (TIA). Renal failure (RF) was defined as chronic kidney disease with estimated glomerular filtration rate [eGFR] below 60 ml/min/1.73 m^2^.

Dyslipidemia was defined as a history of diagnosed and treated hypercholesterolemia or/and hypertriglyceridemia. Heart failure diagnosis was based on the hospitalization history or typical symptoms/signs and hypertension diagnosis was based on the history of antihypertensive treatment or elevated blood pressures over 140/90 mm Hg.

### Endovascular procedure and angiographic evaluation

All patients qualified for repeated endovascular treatment received dual antiplatelet therapy (aspirin 75 mg/d and clopidogrel 75 mg/d or ticagrelor 90 mg BID). Patients with the highest thromboembolic risk qualified to endovascular treatment immediately (ischemic exacerbation, rapid progression of ulceration/necrosis) received additionally low-molecular-weight heparin (LMWH) at prophylactic or half-therapeutic dose before ET with continuation for 4 weeks after the procedure.

All angiograms of primary and re-intervention were independently analyzed off-line by 2 experienced investigators (TN, MK) unaware of the fibrin clot data. All treated segments were analyzed carefully for the presence of unfavorable outcome including dissection limiting vessel flow, peripheral emboli or stent under expansion. Stenosis was defined as significant based on visual inspection or when the degree of stenosis measured with Quantitative Vascular/Coronary Angiography (QVA/QCA) software (Siemens, Germany) was > 50% of lumen diameter [21].

### Postprocedural follow-up

During follow-up after the primary procedure clinical improvement or worsening of the treated limb were evaluated in all patients. Non-invasive tests were also performed. Ankle-brachial index, toe-brachial index (TBI) assessment and duplex ultrasound imaging were performed at 1, 3, 6 and 12 months after primary ET. Regardless of clinical signs and symptoms the initial diagnosis of the restenosis was made using non-invasive tests—primarily ultrasound imaging. Patients with restenosis in the previously treated vessel in ultrasound examination were qualified for angioplasty. Digital subtraction angiography (DSA) which finally confirmed the diagnosis of restenosis was done during the re-PTA procedure.

The restenosis was defined as a reoccurrence of the narrowing of the lumen of a successfully treated vessel segment with more than 50% of artery diameter reduction in DSA.

Patients and their families were instructed about pharmacological treatment after the procedure. They were also encouraged to stop smoking.

The composite endpoint included re-intervention because of restenosis, major amputation and cardiovascular death.

### Laboratory investigations

Blood for the laboratory tests was taken in the postprocedural follow-up period up to 12 months after index PTA both in patients with restenosis and in the control group.

In the CLI patients time of blood sampling after the primary procedure was variable and was associated with the occurance of restenosis. The mean time of blood sampling in this group was the same as the mean time of the re-intervention (22–23 weeks after the primary PTA). In the control group blood samples were taken 3 or 6 months after PTA during the follow-up visit. Blood was drawn from an antecubital vein with minimal stasis after an overnight fast, between 7 and 10 AM. In patients receiving LMWH the blood was collected over 12 h after the last injection.

Blood cell count, creatinine, glucose were assayed by routine laboratory techniques. eGFR was calculated according to the Modification of Diet in Renal Diseases (MDRD) study equation. Fibrinogen was determined using the Clauss method and hs-CRP by the latex nephelometry (Siemens, Marburg, Germany). Commercially available immunoenzymatic assays were used to determine tissue-type plasminogen activator antigen (t-PA:Ag), plasminogen activator inhibitor-1 antigen (PAI-1:Ag) (both American Diagnostica, Stamford, CT, USA) and thrombin activatable fibrinolysis inhibitor antigen (TAFIa/ia) (Imubind TAFIa/ai antigen ELISA; American Diagnostica). Von Willebrand factor (vWF) antigen was measured with immunoturbidimetry using the STA Liatest kit (Diagnostica Stago, Asniéres, France). Plasminogen and α_2_-antiplasmin were measured by chromogenic assays (STA Stachrom antiplasmin and STA Stachrom plasminogen, Diagnostica Stago, Asniéres, France).

Thrombin generation was assessed using the Calibrated Automated Thrombogram (Thrombinoscope BV, Maastricht, the Netherlands) according to the manufacturer’s instructions in the 96-well plate fluorometer (Ascent Reader, Thermolabsystems OY, Helsinki, Finland) [22]. The area under the curve (AUC), as a measure of the endogenous thrombin potential (ETP), expressed the dynamic of thrombin generation in the plasma sample after addition of TF. Each plasma sample was analyzed in duplicate. All intra-assay and inter-assay coefficients of variation were below 7%.

### Plasma fibrin clot analysis

In citrated plasma (vol/vol 9:1 of 3.2% sodium citrate), the following variables describing plasma clot structure and lysability were determined in duplicate by technicians blinded to the origin of the samples (intra-assay and inter-assay coefficients of variation, 5–7%).

### Clot permeability

Permeation properties of fibrin clots were investigated as previously described [23]. At a glance, after incubation of 20 mmol/L calcium chloride, 1 U/mL human thrombin (Sigma, St. Louis, MO) and the citrated plasma, tubes containing the clots were connected to a reservoir of a buffer (0.01 MTris, 0.1M NaCl, pH 7.5) and its volume flowing through the gels was measured within 60 min. A permeation coefficient (K_s_), indicating the pore size, was calculated from the equation: K_s_ = Q × L × η / t × A × Δp, where Q is the flow rate in time t, L is the length of a fibrin gel, η is the viscosity of liquid (in poise), A is the cross-sectional area (in cm^2^), and Δp is a differential pressure (in dyne/cm^2^).

### Clot lysis time

Clot lysis time (CLT) was determined as described [24, 25]. Briefly, the mixture of 10000-diluted human tissue factor (Innovin, Siemens, Marburg, Germany), 12 µmol/l phospholipid vesicles and 60 ng/ml recombinant t-PA (Boehringer Ingelheim, Ingelheim, Germany) was transferred to a microtitre plate and its turbidity was measured at 405 nm at 37 °C. CLT was defined as the time from the midpoint of the clear-to-maximum-turbid transition, which represents clot formation, to the midpoint of the maximum-turbid-to-clear transition representing the clot degradation.

### Statistical analysis

The study was powered to have a 90% chance of detecting a 10% difference in CLT using a p-value of 0.05, based on the values of CLT. In order to demonstrate such a difference or greater, 32 patients were required in each group. In turn, to demonstrate such a difference in K_s_ using a p-value of 0.05, at least 31 patients were required in each group.

Continuous variables are expressed as mean ± SD or otherwise stated (as median with IQR). Continuous variables were checked for normality using the Shapiro–Wilk test and compared by Student’s t-test (with correction for unequal variances if appropriate) when normally distributed or by the Mann–Whitney or Wilcoxon tests for non-normally distributed variables. The Pearson or Spearman rank correlation coefficients were calculated to test the association between two variables with a normal or non-normal distribution, respectively. Odds for the 1st quartile of K_s_ compared to the 4th and for the 4th quartile of CLT compared to the 1st quartile were calculated using simple logistic regression. Multiple logistic regression models were constructed stepwise backward from variables with a *p*-value < 0.2 from simple regression models. Bayesian Information Criterion (BIC) was used as a target.

A *p*-value < 0.05 was considered statistically significant. All calculations were done with JMP, version 13.1 (SAS Institute Inc., Cary, NC, USA).

## Results

### Patient characteristics

**Fig. 1:**
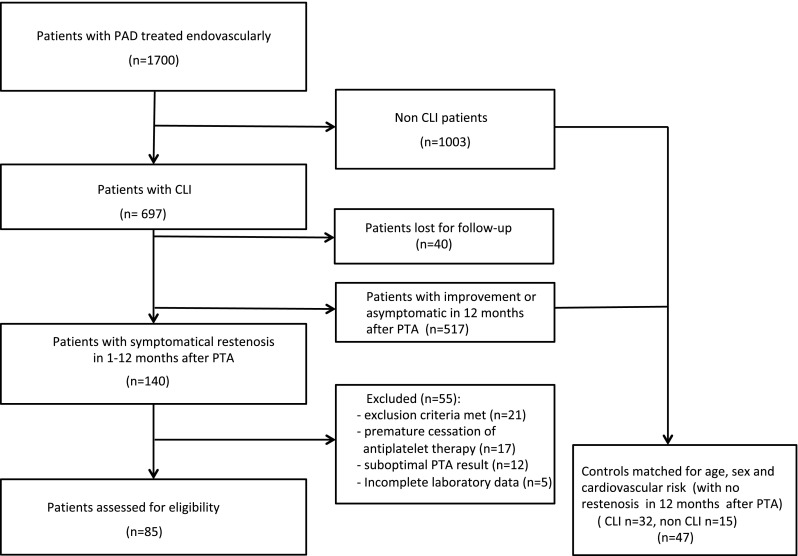
Study patients recruitment and selection diagram

A total of 85 patients with in-stent restenosis/re-occlusion after PTA entered the final analysis (Fig. 1).

Characteristics of patients with restenosis and control subjects were presented in Table 1.

**Table 1:** Characteristics of CLI patients with restenosis and control subjects

	Restenosis group (n = 85)	Controls (n = 47)	*p* value
Age, years	69.65 ± 10.36	66.89 ± 8.46	0.14
Male gender, n (%)	49 (57.65)	33 (70.21)	0.15
BMI, kg/m^2^	25.49 ± 4.91	26.26 ± 3.91	0.13
Current smoking, n (%)	55 (64.71)	35 (74.47)	0.25
Dyslipidemia, n (%)	53 (62.35)	26 (55.32)	0.43
Diabetes, n (%)	38 (44.71)	23 (48.94)	0.64
Hypertension, n (%)	73 (85.88)	42 (89.36)	0.57
Heart failure, n (%)	18 (21.18)	5 (10.64)	0.13
CAD, n (%)	35 (41.18)	17 (36.17)	0.57
MI	20 (23.53)	9 (19.15)	0.56
PCI	13 (15.29)	7 (14.89)	0.04
CABG	5 (5.88)	1 (2.13)	0.42
CVD, n (%)	15 (17.65)	2 (4.26)	0.027
RF, n (%)	25 (29.41)	5 (10.64)	0.013
PAD characteristic			
Rutherford class			
3, n (%)	0 (0.00)	15 (31.91)	< 0.001
4, n (%)	39 (45.88)	8 (17.02)	
5, n (%)	40 (47.06)	18 (38.30)	
6, n (%)	6 (7.06)	6 (12.77)	
ABI	0.28 ± 0.46	0.64 ± 0.36	
TBI	0.05 ± 0.10	0.21 ± 0.19	
Treated segment			
Ilio-femoral, n (%)	9 (10.59)	10 (21.28)	
Femoro-popliteal, n (%)	44 (51.76)	17 (36.17)	0.12
Femoro-popliteal and/or infrapopliteal, n (%)	32 (37.65)	20 (42.55)	
Time to restenosis (weeks)	20.75 ± 13.59	-	-
Time to re-intervention, (w)	23.21 ± 14.17	-	-
Medications, n (%)			
Statins	85 (100)	47 (100)	1.0
Aspirin	85 (100)	47 (100)	1.0
ACEI/ARB	59 (69.41)	32 (68.09)	0.87
BB	44 (52.38)	22 (46.81)	0.54
Calcium antagonists	26 (30.59)	16 (34.04)	0.68
LMWH	47 (55.29)	0 (0.00)	< 0.001
P2Y_12_	84 (98.82)	13 (27.66)	< 0.001
PPI	73 (85.88)	19 (40.43)	< 0.001

Values are given as mean ± SD or number (percentage)

*BMI* body mass index; *CAD* coronary artery disease; *MI* myocardial infarction; *PCI* percutaneous coronary intervention; *CABG* coronary by-pass graft; *CVD* cerebrovascular disease; *TIA* transient ischemic attack; *RF* renal failure; *ABI* ankle/brachial index; *TBI* toe/brachial index; *ASA* acetylsalicylic acid; *ACEI* angiotensin-converting enzyme inhibitor; *ARB* angiotensin receptor blocker; *BB* beta-receptor blocker; *LMWH* low molecular weight heparin; *P2Y*_*12*_ P2Y_12_ receptor inhibitor; *PPI* proton pump inhibitor

84 patients with restenosis (98.7%) had at least one stent implanted during the primary procedure and one patient (1.7%) had balloon angioplasty. During re-intervention 72 (84.7%) patients were treated with stent implantation and 13 (15.3%) with balloon angioplasty alone.

The mean time of restenosis occurrence was 21 weeks (3–50 weeks) and the re-intervention time in most patients (62.4%) was 2–3 weeks longer (*p* = 0.19). 32 patients (37.6%) required immediate intervention.

Both ABI and TBI were lower in the restenosis group. There was no significant difference with regard to the treated vascular segment of the affected limb.

Both groups did not differ in baseline routine laboratory investigations except for lower hemoglobin and eGFR in the restenosis group (Table 2).

**Table 2:** Laboratory parameters and fibrin clot properties measured in patients with restenosis and controls

	Restenosis group (n = 85)	Controls (n = 47)	*p* value
Creatinine, umol/l	87.58 ± 48.98	76.27 ± 23.6	0.076
eGFR, ml/min/1.73 m^2^	81.46 ± 33.96	94.17 ± 26.9	0.024
Hemoglobin, g/l	12.78 ± 1.71	13.85 ± 1.17	< 0.001
Platelets, x10^3^/ul	231.35 ± 80.84	215.96 ± 43.62	0.16
Fibrinogen, g/l	3.84 ± 1.17	3.69 ± 0.63	0.87
CRP, mg/l	5.0 (1.74; 15.40)	5.0 (5.0; 8.2)	0.43
PAI-1, ng/ml	31.1 (25.45; 38.5)	31.8 (28.9; 34.9)	0.62
ETP (nM.min)	1568 (1445; 1691.5)	1441 (1362; 1568)	< 0.001
vWF %	207 (167.5; 239.5)	180 (145; 204)	< 0.001
TAFI ia/a %	96 (85; 107)	100 (94; 108)	0.03
Plasminogen (%)	105 (90.5; 121.5)	105 (97; 119)	0.98
α_2_-antiplasmin (%)	112.51 ± 17.01	109.77 ± 17.76	0.22
tPA Ag, ng/ml	10.81 ± 2.83	10.16 ± 1.98	0.12
*K*_s_, 10^−9^cm^2^	6.38 ± 0.85	6.98 ± 0.79	< 0.001
CLT, min	107.24 ± 22.93	95.40 ± 15.46	0.003

Values are given as mean ± SD or median (IQR)

*eGFR* estimated glomerular filtration rate; *CRP* C-reactive protein; *PAI-1* plasminogen activator inhibitor-1; *ETP* endogenous thrombin potential; *vWF* von Willebrand factor; *TAFI* thrombin activatable fibrinolysis inhibitor; *tPA* tissue-type plasminogen activator; *K*_*s*_ permeability coefficient; *CLT* clot lysis time

After adjustment for severity of PAD (both groups with CLI before primary PTA), the clinical differences observed initially between the whole control group and restenosis group, as well as laboratory parameters differentiating both groups remained still significant (Table 4S, Table 5S).

### Hemostatic variables

Higher thrombin generation (+ 7.9%) was observed in patients with restenosis (Table 2). ETP values in this group positively correlated with fibrinogen and CRP (*r* = 0.51, *p* = 0.0005 and *r* = 0.3, *p* = 0.003 respectively). Moreover, 14.2% higher vWF antigen levels were found in the restenosis group (Table 2). Among fibrinolysis proteins, active TAFI levels were 5% lower also in the restenosis group (*p* = 0.03).

**Fig. 2:**
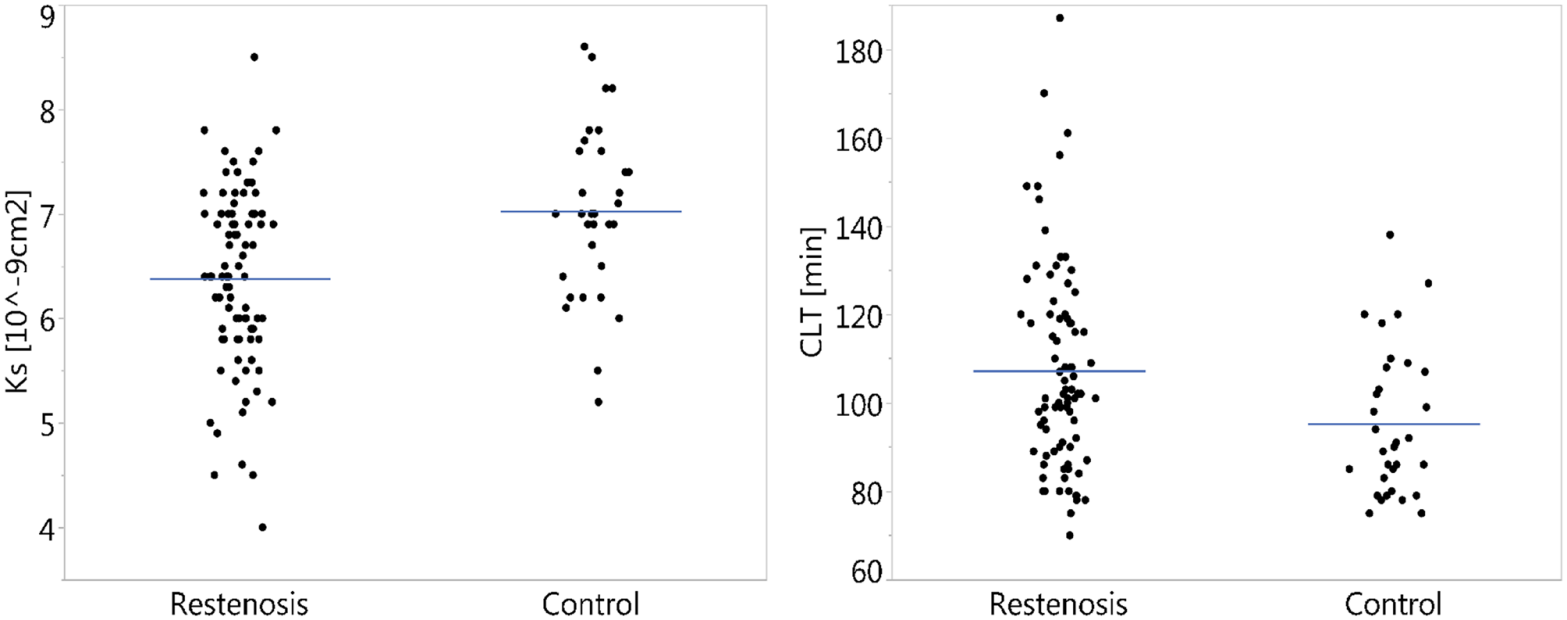
Scatter plots of the permeability coefficient (*K*_s_) and clot lysis time (CLT) measurements in patients with restenosis and control group. Lines represent mean values for both parameters, p < 0.005

Patients with CLI and restenosis had reduced clot permeability (− 9.5%) and prolonged CLT (+ 12.4%) compared with the control group (Fig. 2). K_s_ and CLT showed strong associations with fibrinogen (*r* = − 0.55, *p* < 0.0001 and *r* = 0.56, *p* < 0.001, respectively). CRP correlated with K_s_ (r = − 0.36, *p* = 0.0016) and CLT (*r* = 0.32, *p* = 0.047). The univariate logistic analysis for K_s_ and CLT in the restenosis group is shown in Table 3.

**Table 3:** Univariate logistic analysis. Odds for lower quartile compared to higher quartile of *K*_s_ and odds for upper quartile compared to lower quartile of CLT in the restenosis group

	*K*_s_		*CLT*	
Variable	*OR* (95%CI)	*p* value	*OR* (95%CI)	*p* value
Age	1.00 (0.94; 1.07)	0.89	1.03 (0.97; 1.11)	0.31
BMI	0.95 (0.82; 1.07)	0.40	0.88 (0.75; 1.02)	0.11
eGFR	1.01 (0.99; 1.03)	0.46	0.99 (0.97; 1.01)	0.31
Hematocrit	0.97 (0.84; 1.11)	0.62	0.97 (0.84; 1.10)	0.60
PLT	1.00 (0.99; 1.01)	0.86	1.00 (1.00; 1.01)	0.48
Fibrinogen	8.90 (2.90; 49.01)	0.002	2.66 (1.43; 6.04)	0.007
CRP	1.12 (1.04; 1.23)	0.009	1.05 (1.00; 1.13)	0.11
ABI	0.99 (0.19; 3.95)	0.98	0.37 (0.04; 1.82)	0.31
TBI	1.09 (0.99; 1.2)	0.09	0.99 (0.89; 1.11)	0.95
*K*_**s**_	–	–	0.06 (0.01; 0.24)	0.001
CLT	1.10 (1.05; 1.18)	0.001	–	–
PAI-1	1.17 (1.07; 1.31)	0.002	1.39 (1.19; 1.76)	0.0005
ETP	1.02 (1.01; 1.03)	0.004	1.01 (1.01; 1.02)	0.0008
vWF %	1.01 (0.99; 1.02)	0.39	1.01 (1.00; 1.03)	0.19
TAFI	1.05 (1.01; 1.11)	0.044	1.06 (1.02; 1.13)	0.02
Plasminogen	0.95 (0.90; 0.99)	0.02	0.97 (0.93; 1.01)	0.11
α_2_-antiplasmin	0.99 (0.94; 1.03)	0.48	0.98 (0.95; 1.02)	0.37
tPA	1.03 (0.83; 1.28)	0.77	0.96 (0.77; 1.19)	0.69
AH	0.14 (0.02; 0.75)	0.02	0.71 (0.12; 3.67)	0.71
CAD	0.9 (0.24; 3.26)	0.87	1.47 (0.44; 5.06)	0.67
MI	1.75 (0.36; 8.69)	0.48	1.54 (0.42; 5.81)	0.51
AF	0.49 (0.02; 4.24)	0.53	4.71 (0.62; 96.81)	0.14
CHF	6.9 (1.33; 53.03)	0.02	4.5 (1.09; 23.57)	0.03
DM	0.26 (0.06; 0.98)	0.047	1.21 (0.36; 4.16)	0.75
CVD	0.89 (0.75; 1.33)	0.07	1.0 (0.24; 4.25)	1.0
Smoking	1.69 (0.43; 7.45)	0.45	0.63 (0.15; 2.40)	0.49
RF	0.30 (0.04; 1.46)	0.14	0.78 (0.19; 3.13)	0.72
Statin	0.08 (0.001; 0.86)	0.02	0.32 (0.002; 6.31)	0.36
LMWH	0.67 (0.18; 2.37)	0.52	3.33 (0.95,12.71)	0.05
P2Y_12_	4.76 (1.31; 22.9)	0.02	3.08 (0.96; 11.08)	0.06

^a^In comparison to patients without mentioned co-morbidities and medical therapy

Abbreviations: see Tables 1 and 2

CLT inversely correlated with ABI (*r* = − 0.21) and TBI (*r* = − 0.21) in all 132 patients (*p* < 0.05).

In the control group correlations between fibrinogen concentrations and *K*_s_ (*r* = − 0.55, *p* < 0.001) as well as CLT (*r* = 0.34, *p* = 0.03) were also observed.

### Follow-up

During a mean follow-up of 24.3 months (range, 12–36 months), 54 patients (63.5%) with CLI and restenosis reached a composite endpoint including re-intervention, amputation and death. 18 patients (21.2%) have died—mostly of cardiovascular events (heart failure, acute coronary syndrome). 38 patients (44.7%) had at least one re-intervention but only five patients lost the limb (5.8%). In the control group one patient died (2.1%), 8 underwent recurrent PTA because of restenosis (17%) and none had an amputation.

Patients with restenosis and worse outcomes had more frequent RF, smoking history, concomitant CAD and CVD. No differences in fibrin clot properties and other laboratory variables in this group were observed.

## Discussion

This study demonstrates that altered plasma clot properties and impaired susceptibility to lysis are associated with the history of restenosis within the first year of endovascular treatment in PAD patients with CLI. In patients with CLI and restenosis plasma fibrin clots are less permeable and more resistant to lysis compared to those PAD patients who did not experience restenosis after ET. These findings extended our knowledge on the role of prothrombotic clot features and hypolysibility in advanced PAD.

Our results are consistent with previous investigations in patients with PAD without CLI [17, 18, 20]. While fibrin clots presented more prothrombotic features in CLI patients with restenosis, both groups did not differ according to main inflammatory determinants of clot characteristics, namely fibrinogen and CRP. However, increased levels of fibrinogen and CRP correlated with lower clot permeability and prolonged lysis, which is consistent with data from previous studies [14, 26].

In our data interpretation, several confounding factors should be addressed. We demonstrated that the involvement of unfavorable fibrin clot properties in patients with CLI and restenosis is similar to that reported previously in individuals with re-occlusion (stent thrombosis) following stent implantation in the coronary arteries [25, 27]. However the mechanism of in-stent restenosis remains different than in-stent thrombosis. Most of the known causes of restenosis produce acute or late stent thrombosis where incomplete endothelialization and persistent fibrin-rich thrombi play a crucial role [28]. It is difficult to determine what is the contribution of thrombosis and inflammatory-related neointimal proliferation in restenosis among patients with PAD.

We also observed increased vWF especially in patients of the restenosis group. This marker was found as the independent predictor of fibrin-rich intracoronary thrombus presence in patients with acute MI [29]. Moreover, the extent of vWF release in patients with acute coronary syndrome was an independent predictor of adverse clinical outcome [30]. Although previous observations of the association of vWF and restenosis in PAD are not consistent [31, 32], in the current study elevated levels of vWF in PAD patients could indicate on endothelial cell dysfunction and its contribution to restenosis process in PAD.

Thrombin generation was higher in CLI patients with restenosis and strongly correlated with fibrin clot parameters. Moreover, ETP constituted one of the independent predictors for low *K*_s_ values and high CLT values. This finding is consistent with observations that higher thrombin concentrations produce less permeable clots, composed of a dense fiber network resistant to lysis [33]. Elevated ETP could be involved in atherosclerosis and progression of PAD in part through fibrin-mediated mechanisms. Although in many studies in patients with CAD and fewer in patients with PAD thrombin generation (in vivo) and ETP (ex vivo) were altered, it is not clear how important is the relationship between high thrombin generation and atherosclerosis [34].

Patients were receiving antiplatelet drugs, and it is known that aspirin positively affects clot structure [35]. In our study altered plasma clot properties with more pronounced negative characteristics in the restenosis group were apparent despite aspirin and clopidogrel administration. The beneficial effect of the statins on fibrin properties has also been described [36]. However, all patients in this study were taking statins and the impact of these medications could not have been assessed.

The choice of a 10% difference in CLT or clot permeability as biologically important deserves a comment. We chose this difference based on our praevious studies using a similar methodology [37, 38]. Because of synergistic actions of multiple prothrombotic alterations 10–20% differences could be sufficient to modulate the hypercoagualable state. In our recent cohort study we reported that similar differences in fibrin clot properties have a prognostic value in terms of the risk of recurrent thromboembolism during follow-up [38].

Further studies are needed to validate clinical relevance of our findings among patients with CLI.

### Limitations

This study has several limitations. First, the number of patients was limited, though the study was adequately powered however, the subgroup analysis especially with long outcomes should be interpreted with caution. Secondly, we did not analyze a potential impact of blood cells and platelets on fibrin clot structure/function. Third, other fibrin clot modifiers including oxidative stress and homocysteine have not been investigated in the current study either [18]. Although fibrin parameters can be changed by medical treatment, we did not expect any differences of the impact of such management on these parameters since all patients and controls were treated similarly. The important difference with antiplatelet and anticoagulation treatment was associated with the re-PTA procedure. Before endovascular procedure almost all CLI patients were taken P2Y_12_ inhibitors and half of them received LMWH (Table 1). We did not measure anti-FXa activity before blood sampling to confirm that the patients receiving LMWH had no residual anticoagulant effects. However, > 12 h after the last injection, such effects are rather unlikely.

Finally, an important limitation is the way of recruitment. All the patients were recruited after the primary endovascular procedure with a 12 months follow-up period. To achieve an adequate number of patients in the control group, we enrolled some patients with severe claudication (32%), what influenced on differences in PAD characteristic between analyzed groups and could have an influence on some clinical outcomes. However after adjustment for the severity of PAD (both restenosis and control group with CLI), the clinical and laboratory differences observed initially between the whole control group and restenosis group remained significant.

Our findings cannot be easily extrapolated to patients with severe comorbidities, who were excluded from our study, particularly those with end-stage renal failure shown to alter plasma fibrin properties unfavorably [15, 16].

## Conclusions

Our study demonstrates that fibrin clots in patients with CLI who experienced restenosis despite optimal endovascular and pharmacological therapy are more prothrombotic involving denser fiber meshwork relatively resistant to lysis compared with those with good outcomes over a 1 year follow-up. However, because of the complexity of interactions between cardiovascular risk factors, coagulation proteins, platelets function and pharmacotherapy, our observation does not allow to determine which CLI patients with impaired fibrin clot properties would develop poor outcomes. Further studies are required to confirm hypothetical benefits of prolonged anticoagulant therapy in this subgroup of patients.

## Electronic supplementary material

Below is the link to the electronic supplementary material.

Supplementary material 1 (DOCX 86 KB)
