# Supplementary material for: Restenosis is associated with prothrombotic plasma fibrin clot characteristics in endovascularly treated patients with critical limb ischemia
Source: J Thromb Thrombolysis. 2019 Feb 14;47(4):540–9. doi: 10.1007/s11239-019-01826-9 (PMC6476846; doi:10.1007/s11239-019-01826-9)
Supplement: Supplementary file 1 — Supplementary material 1 (DOCX 86 KB) [file 11239_2019_1826_MOESM1_ESM.docx]

**SUPPLEMENTARY MATERIAL**

**Table 4S**. Characteristics of CLI patients with restenosis and respective control CLI subjects

|  | **Restenosis group**  **(n=85)** | **Controls**  **(n=32)** | ***p* value** |
| --- | --- | --- | --- |
| Age, years | 69.65 ±10.36 | 66.96 ±9.25 | 0.18 |
| Male gender, n (%) | 49 (57.65) | 22 (68.75) | 0.37 |
| BMI, kg/m^2^ | 25.49 ±4.91 | 26.57 ±4.32 | 0.24 |
| Current smoking, n (%) | 55 (64.71) | 22 (68.75) | 0.84 |
| Dyslipidemia, n (%) | 53 (62.35) | 20 (62.5) | 0.84 |
| Diabetes, n (%) | 38 (44.71) | 18 (56,25) | 0.36 |
| Hypertension, n (%) | 73 (85.88) | 27 (84.3) | 0.79 |
| Heart failure, n (%) | 18 (21.18) | 4 (12.5) | 0.42 |
| CAD, n (%) | 35 (41.18) | 13 (40.6) | 0.87 |
| CVD, n (%) | 15 (17.65) | 2 (6.25) | 0.20 |
| RF, n (%) | 25 (29.41) | 3 (9.37) | 0.04 |
| PAD characteristic:  Rutherford class 4, n (%)  5, n (%)  6, n (%)  ABI  TBI | 39 (45.88)  40 (47.06)  6 (7.06)  0.28 ±0.46  0.05 ±0.10 | 8 (25.0)  18 (56.25)  6 (18.75)  0.65 ±0.42  0.16 ±0.17 | 0.049  < 0.0001  0.002 |
| Statins, ASA n (%)  LMWH, n (%)  P2Y_12_ , n (%)  PPI, n (%) | 85 (100) | 32 (100) | 1 |
|  | 47 (55.29) | 0 (0.00) | < 0.0001 |
|  | 84 (98.82) | 10 (31.25) | < 0.0001 |
|  | 73 (85.88) | 15 (46.87) | < 0.0001 |

Abbreviations: see table 1

Values are given as mean ± SD or number (percentage).

**Table 5S.**

Selected laboratory parameters and fibrin clot properties of patients with restenosis and controls (both groups with CLI)

|  | **Restenosis group (n=85)** | **Controls**  **(n=32)** | ***p* value** |
| --- | --- | --- | --- |
| CR, umol/l | 87.58 ±48.98 | 73.37 ±18.35 | 0.02 |
| eGFR, ml/min/1.73m^2^ | 81.46 ±33.96 | 96.93 ±27.94 | 0.01 |
| Fibrinogen, g/l | 3.84 ±1.17 | 3.77 ±0.64 | 0.7 |
| PAI-1, ng/ml | 32.54 ±7.95 | 32.07 ±3.97 | 0.68 |
| ETP (nM.min) | 1579.98 ±185.19 | 1461.4 ±158.35 | 0.001 |
| vWF % | 205.08 ±50.96 | 181.37 ±38.14 | 0.008 |
| *K*_s_, 10^-9^cm^2^ | 6.38 ±0.85 | 7.02 ±0.81 | 0.0004 |
| CLT, min | 107.24 ±22.93 | 95.28 ±16.62 | 0.0027 |

Abbreviations: see table 1

Values are given as mean ± SD.
